# Assessment of the Relationship of Depression With Tobacco and Caffeine Use Among University Students: A Cross-Sectional Study

**DOI:** 10.7759/cureus.19098

**Published:** 2021-10-28

**Authors:** Omar A Safarini, Hamdallah Taya, Yara Abu Elhija, Marah Qadous, Ahmad Farhoud, Ammar Thabaleh, Abdulsalam Khayyat, Zaher Nazzal, Ahmad M Abuhassan, Nesma Ghanim, Fayez Mahamid, Rayyan Al Ali, Basma Damiri

**Affiliations:** 1 Medicine, An-Najah National University, Nablus, PSE; 2 Biomedical Sciences, An-Najah National University, Nablus, PSE; 3 Community and Family Medicine, An-Najah National University, Nablus, PSE; 4 Neurology, An-Najah National University, Nablus, PSE; 5 Clinical Psychology, An-Najah National University, Nablus, PSE; 6 Forensic Medicine, An-Najah National University, Nablus, PSE; 7 Medicine and Health Sciences, An-Najah National University, Nablus, PSE

**Keywords:** risk, psychostimulants, cognitive enhancers, energy drinks consumption, caffeine intoxication, depression, addiction

## Abstract

Background

University students are at a higher risk of using cognitive enhancers and psychoactive substances. Depression is associated with a noticeable decline in academic performance and can increase the risk of substance use. Due to sociopolitical issues, the use of cognitive enhancers and psychoactive substances among Palestinians has spread in the last decade. However, depression among tobacco and caffeine users remains underrecognized and neglected.

Methodology

A self-administrated questionnaire and the Beck Depression Inventory were used to assess the association of depression and the use of cognitive enhancers and psychoactive substances among university students at An-Najah National University in 2020.

Results

The response rate to the questionnaires was 78.8% (n = 1,051; 38.8% males, 61.2% females). The overall prevalence of depression was high (30.6% males, 34.7% females). The prevalence of cigarette smoking (39.2% males, 3.9% females), waterpipe smoking (43.1% males, 21.6% females), energy drink consumption (59.6% males, 29.7% females), coffee consumption (85.5% for each gender), tea consumption, and chocolate consumption was high, with significant differences in accordance to gender and academic fields. The multinomial logistic regression results revealed that cigarette smokers were more likely to have a higher risk of severe (odds ratio [OR] = 4.5, p = 0.001), moderate (OR = 3.27, p < 0.001), and mild depression (OR = 2.24, p = 0.002) than non-smokers. Severe depression was less prevalent among medical students than health sciences and non-medical students (OR = 0.215, p = 0.015). Moreover, males were less likely to have moderate (OR = 0.5, p = 0.012) and mild (OR = 0.48, p = 0.001) depression than females.

Conclusions

Overall, the results of this study revealed the high prevalence of depression and the detrimental effects of smoking on students. Moreover, the findings suggest the urgent need to address depression and risk factors among Palestinian university students by educating them about mental health, identifying high-risk students, and offering easily accessible psychological help. Further, it is crucial to broaden the focus of studies to include students from various academic fields instead of focusing on medical students.

## Introduction

Tobacco smoking and energy drinks (EDs) consumption are widespread in West Bank, Palestine [1-6]. Similar to other countries in the Mediterranean region, the prevalence of tobacco smoking is expected to increase in the coming decades [7]. Moreover, several alternative tobacco products (ATPs), including waterpipes and electronic cigarettes (e-cigarettes or vapes), are now available in addition to traditional cigarettes [1,4,5,8]. Despite the knowledge of health problems associated with tobacco smoking, its prevalence has reached an alarming rate among Palestinian university students [1,3,4]. In addition to tobacco smoking, EDs have become nearly ubiquitous on university campuses in West Bank [1,9].

Depression has been associated with a noticeable decline in academic performance and can increase the risk of substance use [10,11]. University students experience higher rates of depression than the general population [12]. Several studies have demonstrated the association between caffeine and nicotine consumption and depression. In a previous study, coffee, tea, and chocolate were found to have protective effects against depression [13,14], while ED consumption was negatively associated with depression [15]. Additionally, smoking is associated with ED consumption and depression [16,17]. Clinical evidence suggests that women are more susceptible to anxiety disorders, and, consequently, tend to smoke to cope with stress more than men [18]. According to a previous study, medical students suffer from higher stress levels than the general population and students in other academic fields [19]. They tend to suffer from depression during their training due to high academic demands and psychosocial pressure [20]. Therefore, medical students are likely to use cognitive enhancers such as caffeine, ED, and psychostimulants to improve their physical and cognitive functions [21-23].

While the rate of tobacco smoking and ED consumption continues to be a growing problem among Palestinians, research has not thoroughly investigated the various factors associated with the problem. The increasing prevalence of tobacco smoking and ED consumption and its link with negative behaviors and adverse health outcomes for young adult Palestinians requires attention. Few empirical studies have examined the association between these psychoactive enhancers and depression [24,25]. Therefore, this study aimed to assess the relationship between tobacco and caffeine products and depression among university students at An-Najah National University. Awareness of these problems can improve the effectiveness of smoking cessation programs and decrease the potential consequences among young adults. This study is a part of ongoing research that comprehensively investigates and assesses smoking, caffeine, and other substance use and their relationship with different disorders. The results of this study can help the global medical community better approach depression among university students by understanding the complex matrix of causes and risk factors. This article was presented as a conference abstract at the 2021 National Institute of Drug Abuse organized by the National Institutes of Health held from June 21 to June 24, 2021.

## Materials and methods

A cross-sectional study was conducted between October 2020 and January 2021. Undergraduate students from different academic fields at An-Najah National University (ANNU) were recruited to participate in this study. First, students were recruited by announcements on social media and flyers, including a complete explanation of the research objectives and requirements. From the recruited students, a stratified sampling technique was used in three stages to choose a representative sample from each academic field. In the first stage, a male-to-female ratio of 1:1 was employed. Then, students were stratified following their curricular year from the first to the sixth year. Finally, a proportional classification according to academic specialization was applied: 20% were medical students, 30% were health sciences students, and 50% were from non-medical academic fields.

Students who met the inclusion criteria were asked to complete an offline self-administered questionnaire. The Arabic version of the Beck Depression Inventory (BDI-II) was used to assess depression among the participants [26]. BDI-II is a 21-question, multiple-choice, self-report inventory and is one of the most commonly used tools to evaluate the severity of depression. BDI-II categorizes depression into four subtypes: minimal, mild, moderate, and severe. Each of the 21 items corresponding to a symptom of depression is summed for a single score. There is a four-point scale for each item ranging from 0 to 3. A total score of 0-13 is considered minimal depression, 14-19 is mild depression, 20-28 is moderate depression, and 29-63 is severe depression. Minimal depression refers to people without depression; hence, the total prevalence of depression is a combination of mild, moderate, and severe results. The Arabic version is valid and reliable among undergraduate students in Arabic countries (including Palestine), with an alpha reliability of 0.83 and test-retest reliability of 0.74 [26]. The questionnaire used to assess the practices and patterns of tobacco and caffeine products was used in a previous study [1]. A current substance user was defined as a student who had used the substance in the past 30 days [27]. ED included locally manufactured and imported products available for purchase in Palestine at the time of the study. The academic specialization included medical students (preclinical or clinical students), students studying health sciences (pharmacy, doctor of pharmacy, medical laboratory, radiology, ophthalmometry, speech and audiology, nursing, and occupational therapy), and students from other faculties. Preclinical medical students include students in their first to third curricular years of the medicine program at ANNU, whereas clinical students include students in their fourth to sixth curricular years of the medicine program at ANNU.

SPSS version 22 (IBM Corp., Armonk, NY, USA) was used for data analysis. Kolmogorov-Smirnov test was used to test the normality of the distribution of continuous variables. Differences in the means between groups were assessed using the independent samples t-test and analysis of variance (ANOVA). Pearson’s chi-square test or Fisher’s exact test were used for categorical variables. Multinominal logistic regression analysis evaluated the relative risk by generating odds ratios (ORs) and 95% confidence intervals (CIs) for risk factors. A p-value of less than 0.05 was considered statistically significant.

All study procedures followed the ethical standards of the responsible committee on human experimentation (institutional and national) and the Helsinki Declaration of 1975, revised in 2000. Approval was obtained from the Institutional Review Board at ANNU, Palestine. Informed consent was obtained from all participants. Confidentiality was highlighted in all written and oral communications.

## Results

The overall number of questionnaires distributed was 1,470, of which 1,158 questionnaires were returned, with a response rate of 78.8%. Partially completed BDI-II questionnaires (n = 28) were discarded. Partially completed substance practice and pattern questions (containing more than 5% of missed information, n = 36) were also discarded. In addition, 43 students withdrew from the study. Hence, the final number of enrolled students was 1,051 (38.8% males, 61.2% females). The majority of the students were singles (98.2%), and 50.4% were urban students. In the final sample, 23.1% were medical students, 32.2% were health sciences students, and 44.6% were non-medical students. The majority of students were non-workers (87.5%) (Table 1).

**Table 1:** Sociodemographic and lifestyle characteristics of students (n = 1,051).

	Males n (%)	Females n (%)	Total n (%)
Gender	408 (38.8)	643 (61.2)	1,051 (100)
Study year			
1–2	164 (40.1)	316 (49.1)	480 (45.6)
3–4	128 (31.3)	238 (37.0)	366 (34.8)
5–6	114 (27.9)	89 (13.8)	203 (19.3)
Academic field			
Medicine	122 (30.3)	119 (18.6)	241 (23.1)
Health sciences	126 (31.3)	210 (32.9)	336 (32.2)
Non-medical fields	155 (38.5)	310 (48.5)	465 (44.6)
Residency			
City	189 (46.3)	341 (53.0)	530 (50.4)
Village	203 (49.8)	293 (45.6)	496 (47.2)
Camp	16 (3.9)	9 (1.4)	25 (2.4)
Marital status			
Single	405 (99.3)	627 (97.5)	1032 (98.2)
Non-single	3 (0.7)	16 (2.5)	19 (1.8)
Working status			
Yes	98 (24.0)	33 (5.1)	131 (12.5)
No	310 (76.0)	610 (94.9)	920 (87.5)

Appendix (Table 5) presents the prevalence and practice findings of cognitive enhancers and psychostimulants among students. The prevalence of cigarette smoking was 17.6% (39.2% among males and 3.9% among females, p < 0.001), waterpipe smoking was 30% (43.1% among males and 21.6% among females, p < 0.001), and e-cigarette smoking was 4.7% (8.8% among males and 2% among females, p < 0.001). ED consumption was highly prevalent among students, with a significant increase among males (59.6%) compared to females (29.7%) (p < 0.001). Chocolate was the most prevalent cognitive enhancer used (92.5%), with a significant increase among females (96.6%) compared to males (86%) (p < 0.001). Coffee (85.5%) and tea (84.3%) were the next most commonly used cognitive enhancers, with no differences in consumption between males and females (p > 0.05). Moreover, no significant differences were observed in the initiation age between males and females for all substances, except waterpipe smoking (16.5 years for males and 17.7 for females, p = 0.007) and e-cigarette smoking (18.3 years for males and 18.7 years for females, p = 0.024). Only 1.1% of the students were not using any substance. Regarding concurrent use of different enhancers and psychostimulants, 37.9% of students were using at least three substances, 23.7% were using at least four substances, and 14.1%, 6.2%, and 2.2% of the students were using at least five, six, and seven substances, respectively, at the same time. Moreover, 8.3% of the students (18.4% of males and 1.9% of females) used four substances simultaneously, namely, cigarettes, waterpipes, coffee, and ED. For tobacco smoking, 10.7% smoked cigarettes and waterpipes (23.5% of males and 2.5% of females), and 25.8% smoked at least one of them (35.3% of males and 19.8% of females). In terms of caffeine consumption, 37.3% of the students (53.7% of males and 26.9% of females) used both coffee and ED, while 52.2% of students (37.7% of males and 61.4% of females) used one of them (p < 0.001) (Appendix, Table 5).

Appendix (Table 6) presents the pattern of cognitive enhancers and psychostimulants tested in the study. For daily use, 59.1% smoked cigarettes (67.5% of males and 7.7% of females), 23.6% smoked waterpipes (30.3% of males and 15.3% of females), 13.8% used ED (18.7% of males and 7.7% of females), 50.5% consumed coffee (56.3% of males and 46.9% of females), and 35.2% consumed chocolate (33.2% of males and 36.2% of females) (Appendix, Table 6).

Waterpipe smoking was less prevalent among medical students (22.4%) than students in health sciences (32.7%) and non-medical academic fields (31.6%) (p = 0.015). On the other hand, chocolate consumption was more prevalent among health sciences (94.9%) than in medicine (87.1%) and non-medical academic fields (93.8%) (p = 0.001). No other differences in the prevalence of other substances consumption were observed (p > 0.05) (Table 2).

**Table 2:** Differences in cognitive enhancer and psychostimulant use among different academic fields. *significant values

Substance	Medicine	Health sciences	Non-medical fields	P-value
	n (%)	n (%)	n (%)	
Cigarette	41 (17.0)	68 (20.2)	71 (15.3)	0.184
Waterpipe	54 (22.4)	110 (32.7)	147 (31.6)	0.015*
E-cigarette	12 (5.0)	17 (5.1)	20 (4.3)	0.859
Energy drink	101 (41.9)	147 (43.8)	182 (39.1)	0.414
Coffee	200 (83.0)	288 (85.7)	405 (87.1)	0.335
Tea	204 (84.6)	281 (83.6)	396 (85.2)	0.839
Chocolate	210 (87.1)	319 (94.9)	436 (93.8)	0.001*

Curiosity was the most prevalent motivation for cigarette smoking (53.9%) and ED consumption (47.4%). Most students (59.9%) smoked waterpipes for fun. Other motivations for ED, coffee, and tea consumption were to increase wakefulness and improve vigilance and attention (Appendix, Table 7).

The prevalence of depression among students was 33.1% (95% CI: 30.3-36.1%); 4.3% severe, 8.8% moderate, and 20.1% mild depression. The prevalence of depression was higher among females (34.7%) than males (30.6%), with no significant difference (p = 0.209). Depression was less prevalent among medical students (31.7%) than health sciences students (32.6%) and non-medical students (34.3%) (p = 0.39). Among medical students, preclinical students were more depressed (33.1%) than clinical students (29.6%), with no significant differences (p > 0.05) (Table 3).

**Table 3:** Prevalence of depression and depression severity following the Beck Depression Inventory in accordance to gender, cognitive enhancer/psychoactive substance use, and different academic fields. *Significant values

Category	Depression					
	Total	Severe	Moderate	Mild	Minimum	P-value
	n (%)	n (%)	n (%)	n (%)	n (%)	
Gender						
Males	125 (30.6)	21 (5.1)	33 (8.1)	71 (17.4)	283 (69.4)	0.209
Females	223 (34.7)	24 (3.7)	59 (9.2)	140 (21.8)	419 (65.3)	
Cigarette smokers	86 (46.5)	16 (8.6)	25 (13.5)	45 (24.3)	99 (53.5)	<0.001*
Water pipe smoking	113 (35.9)	13 (4.1)	33 (10.5)	67 (21.3)	202 (64.1)	0.504
E-cigarette smoking	21 (42.9)	3 (6.1)	6 (12.2)	12 (24.5)	28 (57.1)	0.506
Energy drink consumption	157 (36.2)	22 (5.1)	44 (10.1)	91 (21.0)	277 (63.8)	0.266
Coffee consumption	306 (34.0)	35 (3.9)	80 (8.9)	191 (21.2)	593 (66.0)	0.064
Tea consumption	292 (33.0)	32 (3.6)	76 (8.6)	184 (20.8)	593 (67.0)	0.058
Chocolate consumption	323 (33.3)	38 (3.9)	85 (8.8)	200 (20.6)	648 (66.7)	0.121
Academic field						0.391
Medicine (total)	76 (31.7)	3 (1.3)	18 (7.5)	55 (22.9)	164 (68.3)	
Clinical medicine	29 (29.6)	1 (1.0)	6 (6.1)	22 (22.4)	69 (70.4)	
Preclinical medicine	47 (33.1)	2 (1.4)	12 (8.5)	33 (23.2)	95 (66.9)	
Health sciences	109 (32.6)	18 (5.4)	29 (8.7)	62 (18.6)	225 (67.4)	
Non-medical	163 (34.3)	24 (5.1)	45 (9.5)	94 (19.8)	312 (65.7)	

Table 4 presents the multinominal logistic regression model results of the association between levels of depression among students (dependent variable) and the use of cognitive enhancers and psychostimulants (independent variables) adjusted to the study year, gender, and academic field. Cigarette smoking was associated with all levels of depression, that is, severe depression (OR = 4.5, p = 0.001), moderate depression (OR = 3.3, p < 0.001), and mild depression (OR = 2.3, p = 0.002). No associations were found between depression and waterpipe smoking, e-cigarette smoking, and ED, coffee, tea, and chocolate consumption (p ≥ 0.05). Medical students were less likely to have severe depression (OR = 0.215, p = 0.015) than students in health sciences and non-medical fields (Table 4).

**Table 4:** Multinominal logistic regression for the association between the level of depression and cognitive enhancers. Reference groups are minimum depression (no depression), non-substance user, and non-medical academic field. *Significant values

Category	Odds ratio	95% Confidence interval	P-value
Severe depression			
Study year	0.900	0.714-1.134	0.372
Gender			
Male	0.694	0.313-1.540	0.370
Female	1		
Cigarette smoking	4.512	1.866-10.910	0.001*
Waterpipe smoking	0.705	0.329-1.511	0.368
E-cigarette smoking	1.080	0.279-4.173	0.911
Energy drink consumption	1.289	0.632-2.628	0.485
Coffee consumption	0.573	0.261-1.255	0.164
Tea consumption	0.567	0.270-1.189	0.133
Chocolate consumption	0.520	0.196-1.379	0.189
Academic field			
Medicine	0.215	0.062-0.745	0.015*
Health sciences	0.915	0.458-1.832	0.803
Non-medical	1		
Moderate depression			
Study year	0.951	0.813-1.113	0.532
Gender			
Male	0.479	0.269-0.852	0.012*
Female	1		
Cigarette smoking	3.271	1.686-6.346	<0.001*
Waterpipe smoking	1.140	0.673-1.932	0.626
E-cigarette smoking	1.069	0.398-2.869	0.894
Energy drink consumption	1.198	0.719-1.995	0.488
Coffee consumption	1.035	0.533-2.011	0.918
Tea consumption	0.840	0.455-1.550	0.577
Chocolate consumption	0.919	0.377-2.238	0.852
Academic field			
Medicine	0.818	0.451-1.484	0.508
Health sciences	0.785	0.463-1.331	0.368
Non-medical	1		
Mild depression			
Study year	1.005	0.901-1.120	0.930
Gender			
Male	0.508	0.339-0.760	0.001*
Female	1		
Cigarette smoking	2.235	1.356-3.685	0.002*
Waterpipe smoking	1.032	0.700-1.520	0.874
E-cigarette smoking	1.177	0.554-2.502	0.672
Energy drink consumption	1.061	0.737-1.529	0.749
Coffee consumption	1.609	0.948-2.731	0.078
Tea consumption	1.124	0.693-1.825	0.635
Chocolate consumption	1.215	0.592-2.494	0.595
Academic field			
Medicine	1.233	0.826-1.841	0.305
Health sciences	0.903	0.615-1.326	0.603
Non-medical	1		

## Discussion

There are several remarkable findings of this study. First, the overall prevalence of depression among Palestinian university students was high (33.1%), although it was lower than that reported in previous studies from the Gaza strip (37.2%) [28]. While it was within the range of the prevalence of depression among university students reported in a systematic review (10-84.5%), it was higher than the weighted mean prevalence (30.6%) [12]. Moreover, the prevalence was noticeably higher than the general population of West Bank (24.3%) [29]. This difference in prevalence between university students and the general population is consistent with other global studies [12]. Second, the multinomial logistic regression findings of this study revealed the detrimental effects of smoking on students. Being a smoker was highly associated with depression, particularly severe depression. Moreover, according to our results, the overall prevalence of cigarette and waterpipe smoking was high among university students with clear differences in gender and academic fields.

Females tend to smoke to cope with stress more than men as they are more susceptible to anxiety disorders [18,30,31]. In agreement with other studies, depression was more prevalent among female university students (34.7%) than males (30.6%) [12,32]. However, males had a higher prevalence of severe depression than females. Unfortunately, depression often remains undiagnosed and untreated, especially among males [33]. In addition, males are less likely to seek psychological consultation than females, and depressed males are more likely to commit suicide than depressed females [12].

In agreement with previous studies, most students in this study smoked as a coping mechanism for stress or family and social peer pressure [34-36]. Depression has catastrophic effects ranging from low quality of life to somatic symptoms and increased risk of suicide [37,38]. People with depression have specific difficulties stopping smoking and have more severe withdrawal symptoms [39]. Females have lower smoking cessation rates compared to males [30]. Therefore, the high prevalence of smoking among students and its association with depression is concerning. We recommend that healthcare systems provide prevention, treatment, and recovery support services for smokers.

The vast majority of global research concerning depression among university students has been confined to medical students, probably due to the enormous burden of stress and the challenges that medical students face in different stages of education [21,40]. Because of these stressors, medical students are more susceptible to using cognitive enhancers and psychoactive substances to improve their vigilance and attention [41]. However, personal factors such as knowledge, risk perception, attitudes, motivation, and social influence can impact smoking and the desire to quit smoking [5,42]. According to our findings, although the prevalence of depression was high among medical students (31.7%), it was lower than non-medical students (34.3%). In line with previous results, medical students in the clinical stage had the lowest rate of depression (29.6%), which was not different among preclinical students (33.1%) [43,44].

Surprisingly, medical students were five times less likely to have severe depression than other students, while no mild and moderate depression differences were noticed. These findings highlight the need to broaden the scope of such studies to include and compare students from various academic fields instead of only focusing on medical students. Moreover, there is an urgent need to address depression among Palestinian university students by educating them about mental health, identifying high-risk students, and offering easily accessible psychological help.

A previous study targeting medical students in the same university (ANNU) showed that medical students had a total depression prevalence of 43.4%. Among medical students, 9% were severely depressed [45]. These results differ in our study even though the population is the same and the same tool was employed to determine depression (BDI-II). Medical students during their training tend to become depressed due to a high level of academic demand and psychosocial pressure [46]. Several factors may account for this, including daily life stressors and stressors specific to the tedious learning environment [21]. Because our research was conducted during the coronavirus disease 2019 (COVID-19) pandemic, we speculate that a significant part of these differences could be attributed to COVID-19-related quarantine. Consequently, medical students received a blended educational system. The change in the academic structure and lifestyle of medical students could have played a key role in mitigating their stress levels and sequentially reducing the prevalence and severity of depression. Therefore, we recommend conducting a similar study after the COVID-19 pandemic and exploring the differences in results. Additionally, such a comparison will give an insight into the effects of pandemics on the mental health of university students.

In contrast to other studies, there was no association between ED consumption and depression, despite the high prevalence and regular use of caffeine among both genders [15,47-50]. Reasons for the consumption of ED included curiosity, improving cognitive performance, increasing attention span, peer pressure, fun, and relaxation, indicating that ED and caffeine consumption is a habit among Palestinian university students, in general, and males, in specific. This could be a potential health risk. The early initiation age and motivations of use indicate that using these substances preceded depression among university students. Early intervention could be vital to directing students’ life paths away from high-risk behaviors such as nicotine and caffeine consumption. Further studies are recommended to determine if ED consumption among university students is higher than the general population, with a male gender predominance [51].

The use of ED by medical students is controversial. On one hand, medical students could be less likely to use ED as they have more knowledge regarding its side effects than non-medical students [52]. On the other hand, medical students could be more prone to consume ED. ED consumption was high among medical and non-medical students with no significant differences. Student awareness of caffeine intoxication, withdrawal, and dependence should be improved. Universities should take more action to prevent ED consumption among young adults and develop effective prevention and cessation strategies. They need to make more efforts in implementing ED and smoking cessation programs. Policies addressing students’ well-being and mental health and policies that regulate ED consumption may be necessary. Moreover, enacting and enforcing comprehensive smoke-free policies prohibiting smoking in enclosed universities and public places are strongly recommended.

Several limitations should be considered when interpreting the results of this study. First, an anonymous self-report behavioral survey was used instead of more reliable structured interview-based methods due to the stigma surrounding tobacco smoking by women. Self-report questionnaires, generally used for computing smoking statistics, provide underestimated outcomes compared to the actual smoking prevalence, especially among women. Second, the scales used in this study to assess depression cannot confirm a diagnosis. Finally, the lack of mental health assessment by experts made our study valuable only as an initial screening method. However, this study highlighting the detrimental effects of smoking on students prompts the need to broaden the scope of studies to include students from various academic fields instead of focusing on medical students and males.

## Conclusions

Tobacco smoking, ED consumption, and alternative tobacco products are highly prevalent among Palestinian university students. The smoking rates among students with depression are higher than those without depression. Moreover, tobacco smoking among women has become an increasing problem in the West Bank region. Therefore, the rising prevalence of nicotine and caffeine use and their link to adverse health outcomes for Palestinian students requires attention. Awareness of these problems can improve the effectiveness of smoking cessation programs and reduce the risk of potential consequences. Collectively, our results suggest the urgent need to address depression among university students in Palestine by educating them about mental health, identifying high-risk students, and offering easily accessible psychological help.

## Appendices

**Table 5:** The prevalence and practice of cognitive enhancers and psychostimulants tested in the study.

	Males n (%)	Females n (%)	Total n (%)	P-value
Current user				
Cigarettes	160 (39.2)	25 (3.9)	185 (17.6)	<0.001
Waterpipes	176 (43.1)	139 (21.6)	315 (30.0)	<0.001
E-cigarettes	36 (8.8)	13 (2.0)	49 (4.7)	<0.001
Cigarettes and waterpipes	96 (23.5)	16 (2.5)	112 (10.7)	<0.001
Energy drinks	243 (59.6)	191 (29.7)	434 (41.3)	<0.001
Coffee	349 (85.5)	550 (85.5)	899 (85.5)	0.999
Energy drinks and coffee	219 (53.7)	173 (26.9)	392 (37.3)	<0.001
Tea	341 (83.6)	545 (84.8)	886 (84.3)	0.608
Chocolate	351 (86.0)	621 (96.6)	972 (92.5)	<0.001
Cigarettes, waterpipes, energy drinks, and coffee	75 (18.4)	12 (1.9)	87 (8.3)	<0.001
Ex-user				
Cigarettes	5 (3.1)	1 (3.8)	6 (3.2)	<0.001
Waterpipes	10 (5.6)	8 (5.6)	18 (5.6)	<0.001
E-cigarettes	9 (22.0)	3 (18.8)	12 (21.1)	0.292
Energy drinks	10 (4.1)	5 (2.6)	15 (3.4)	<0.001
Coffee	0 (0.0)	6 (1.1)	6 (0.7)	<0.001
Tea	2 (0.6)	3 (0.6)	5 (0.6)	0.944
Chocolate	5 (1.5)	5 (0.8)	10 (1.1)	0.016
Initiation age in years (Mean ± SD)				
Cigarettes smoking	16.8 ± 2.7	17.4 ± 2.3		0.306
Waterpipe smoking	16.5 ± 2.9	17.7 ± 1.9		0.007
E-cigarette smoking	18.3 ± 3.4	18.7 ± 1.0		0.024
Energy drink consumption	15.7 ± 2.6	16.7 ± 2.6		0.998
Coffee consumption	14.5 ± 3.3	15.1 ± 3.3		0.289
Tea consumption	9.6 ± 3.96	10.0 ± 4.3		0.309
Chocolate consumption	6.8 ± 3.7	6.1 ± 3.9		0.416

**Table 6:** The pattern of cognitive enhancers and psychostimulants tested in the study.

Category	Cigarettes	Waterpipes	E-cigarettes	Energy drinks	Coffee	Tea	Chocolate
	n (%)	n (%)	n (%)	n (%)	n (%)	n (%)	n (%)
Daily							
Males	108 (67.5)	54 (30.3)	8 (19.5)	46 (18.7)	184 (56.3)	141 (44.8)	113 (33.2)
Females	2 (7.7)	22 (15.3)	2 (12.5)	15 (7.7)	251 (46.9)	237 (45.6)	216 (36.2)
Several times/week							
Males	20 (12.5)	31 (17.4)	4 (9.8)	82 (33.3)	93 (24.1)	105 (33.7)	144 (37.4)
Females	3 (11.5)	42 (29.2)	1 (6.3)	41 (20.9)	174 (28.0)	178 (34.2)	282 (46.0)
Several times/month							
Males	17 (10.6)	54 (30.3)	7 (17.1)	73 (29.7)	38 (9.8)	51 (16.2)	63 (16.4)
Females	5 (19.2)	29 (20.1)	0 (0.0)	81 (41.3)	80 (12.9)	72 (13.8)	79 (12.9)
Several times/year							
Males	10 (6.2)	29 (16.2)	13 (31.7)	35 (14.2)	12 (3.1)	15 (4.8)	9 (2.3)
Females	15 (57.7)	43 (29.9)	10 (62.5)	54 (27.5)	24 (3.9)	30 (5.8)	5 (2.5)

**Table 7:** Motivations for the usage of cognitive enhancers and psychostimulants tested in this study.

Motivation	Cigarettes smoking	Waterpipe smoking	E-cigarette smoking	Energy drink. consumption	Coffee and tea consumption	Chocolate consumption
	n (%)	n (%)	n (%)	n (%)	n (%)	n (%)
Increase vigilance and attention	50 (27.0)	49 (15.6)	7 (15.0)	155 (35.5)	470 (44.7)	273 (28.0)
Wakefulness	44 (23.7)	74 (23.3)	10 (21.4)	174 (40.1)	370 (35.2)	138 (14.1)
Fun	72 (38.9)	189 (59.9)	16 (32.1)	157 (36.0)	369 (35.1)	418 (42.2)
Relax	52 (28.1)	78 (24.6)	9 (19.2)	60 (13.7)	343 (32.6)	232 (23.8)
Peer pressure	63 (34.0)	151 (47.9)	9 (19.2)	114 (26.3)	282 (26.9)	207 (21.2)
Boredom	56 (30.1)	106 (33.6)	9 (19.2)	62 (14.2)	220 (20.9)	183 (18.7)
Curiosity	100 (53.9)	160 (50.6)	43 (87.9)	206 (47.4)	148 (14.1)	138 (14.1)
Pleasure	61 (32.9)	50 (15.9)	6 (12.8)	46 (10.6)	135 (12.1)	138 (14.1)
Problem escape	75 (40.8)	82 (22.9)	1 (2.1)	38 (12.1)	117 (11.1)	109 (11.2)
Anger	81 (43.7)	58 (18.3)	6 (12.8)	52 (12.1)	122 (11.6)	124 (12.7)
Addiction	64 (34.6)	59 (18.6)	10 (22.4)	38 (8.7)	168 (16.0)	129 (13.2)
I do not know	18 (9.6)	33 (10.3)	10 (22.4)	52 (12.1)	84 (8.0)	105 (10.8)
No reason	22 (11.9)	30 (9.6)	13 (25.7)	64 (14.7)	104 (9.9)	155 (15.8)
COVID-19 quarantine	18 (9.6)	37 (11.6)	6 (12.8)	22 (5.0)	98 (9.3)	106 (10.9)
Losing weight	14 (7.3)	19 (5.9)	3 (6.4)	15 (3.3)	62 (5.9)	26 (2.7)
Others	4 (2.2)	12 (3.9)	7 (15.0)	8 (1.9)	23 (3.2)	31 (3.1)
